# Evaluation and comparison of three virucidal agents on inactivation of Nipah virus

**DOI:** 10.1038/s41598-022-15228-0

**Published:** 2022-07-05

**Authors:** Yi Huang, Shuqi Xiao, Donglin Song, Zhiming Yuan

**Affiliations:** 1 National Biosafety Laboratory, Chinese Academy of Sciences, Wuhan, 430020 Hubei People’s Republic of China; 2 Wuhan Institute of Virology, Chinese Academy of Sciences, Wuhan, 430071 Hubei People’s Republic of China

**Keywords:** Microbiology, Health occupations

## Abstract

Modern human activity is profoundly changing our relationship with microorganisms with the startling rise in the rate of emerging infectious diseases. Nipah virus together with Ebola virus and SARS-CoV-2 are prominent examples. Since COVID-19 and the West African Ebola virus disease outbreak, different chemical disinfectants have been developed for preventing the direct spread of viruses and their efficacy has also been evaluated. However, there are currently no published efficacy studies for the chemical disinfection of Nipah virus. In this study, the virucidal efficacy of three disinfectants (Micro-Chem Plus detergent disinfectant cleaner, FWD and Medical EtOH) against Nipah virus was evaluated in quantitative suspension tests including. Our results showed that the > 4 log reduction achieved for all products in inactivating Nipah virus in 15 s. Even, 19% ethanol was able to inactivate Nipah virus when applied for at least 8 min contact time. Comparative analysis displayed virucidal efficacy of each of the evaluated disinfectants against SARS-CoV-2, Ebola virus and Nipah virus, with only minor differences in working concentrations and contact times required for complete inactivation. We expect that our study can assist in decontamination in healthcare settings and high level biosafety laboratories and can be beneficial to control for emerging enveloped viruses.

## Introduction

Modern human activity is profoundly and irreversibly changing our relationship with microorganisms to far-reaching effect^1,2^. The impact of this altered relationship can be seen by the startling rise in the rate of emergence of novel viral diseases^3^. Climate change, deforestation and anthropogenic factors, such as farming, industrialization and increased global travel, are all creating greater opportunity for human exposure to new pathogens, which may result in the emergence of viral disease^3–5^. Human immunodeficiency virus (HIV), avian influenza, Hendra and Nipah viruses, SARS, MERS and SARS-CoV-2, and Ebola and Marburg viruses are examples, and all of the emerging infectious diseases induced by these pathogens present a serious and increasing threat to public health and global economies. Though the lack of preparedness is multifactorial, the 2014/2015 Ebola virus disease outbreak in West Africa and the ongoing SARS-CoV-2/COVID-19 pandemic shows that the global community remains ill-prepared for these emerging infectious diseases. Viral surveillance or rapid detection, treatment, vaccination and decontamination or improved sanitation are all key component of successful infection prevention and control programs for emerging infectious viral diseases.

Beside SARS-CoV-2, the infectious agent of the current COVID-19 pandemic as a novel coronavirus, serious diseases caused by Ebola virus and Nipah virus are listed in the World Health Organization’s List of Blueprint Priority Diseases due to their potential to cause a public health emergency and due to the absence of efficacious drugs and/or vaccines^6^. Nipah virus is a member of the Henipavirus genus of the *Paramyxoviridae* family, members of which include the human pathogenic viruses Hedra virus, measles virus, mumps virus and human parainfluenza virus, and is a zoonotic virus with a high case fatality rate^7^. It possesses a single-stranded, non-segmented, negative sense RNA genome fully encapsulated by a lipid envelop, envelope proteins, and a total of six genes (N, P, M, F, G and L) which encode 3 envelope structural proteins, nucleocapsid protein, polymerase, and phosphoprotein. The clinical and autopsy findings revealed that the clinical symptoms in an early outbreak were mostly localized to the central nervous system including drowsiness, headache, and some degree of encephalopathy^7^. With different clinical and epidemiological characteristics, the Bangladesh strain responsible for the more recent outbreaks is more commonly associated with respiratory symptoms, human-to-human transmission, and a higher overall case fatality rate (~ 75%), though the genomes of Nipah virus-Bangladesh and Nipah virus-Malaysia are 91.8% homologous^8^. Furthermore, there is growing evidence of rapid adaptation of Nipah virus to other hosts, including pigs and horses and infected humans display varying modes of transmission.

Since the COVID-19 and the West African Ebola virus disease outbreaks, studies have revealed that SARS-CoV-2 and Ebola virus can remain infectious for significant periods of time on multiple surface types in the environment, including plastic, glass, stainless steel, and banknotes, and even personal protective equipment (PPE)^9–12^. There are quite a few preventative measures that have been adopted to limit the spread of these virus, including those based on chemical, heat and radiation treatment^13–17^. However, the data for persistence of Nipah virus under various environmental conditions are limited^18^, and there are currently no published efficacy studies available for the chemical disinfection of Nipah virus, other than some methods for the safe removal and handling of virus infected samples, such as human serum or plasma and infected tissues, from BSL-4 facility^19–21^. The paucity of virucidal efficacy data for Nipah virus and certain other of the World Health Organization’s Priority List viruses has been mentioned also in a recent review by Ijaz et al.^22^.

In the present study, we evaluated the efficacy of two quaternary ammonium (QAC) biocides, including MICRO-CHEM PLUS Detergent Disinfectant Cleaner (MCP) and FWD (a novel, eco-friendly dual quaternary ammonium compound), for inactivating Nipah virus. The virucidal efficacies determined for the dual QAC were compared with that for ethanol, one of the important active ingredients in some chemical disinfectants and used commonly as a low-level disinfectant in healthcare settings for many years. The different concentrations of MCP, FWD and Medical EtOH and contact times from 15 s to 8 min were evaluated in a quantitative suspension test according to Technical Standard for Disinfection of China^23^. We summarized and compared the efficacies of the three virucidal agents for inactivation of Nipah virus, Ebola virus and SARS-CoV-2.

## Results

### Determination of cytotoxicity

In order to determine the minimal dilution of MCP and FWD that does not exhibit cytotoxicity to Vero E6 cell monolayers, four dilution of MCP and FWD (5%) and two dilution of Medical EtOH® were performed in cell culture medium. The MCP, FWD and Medical EtOH® dilutions were added to Vero E6 cell mono-layers in three replicates and incubated at 37 °C with 5% CO_2_. Each treated cell monolayer was examined by light microscopy to assess cytotoxicity daily. The results showed that no obvious cytotoxicity was demonstrated in cell culture media at a 1:500 dilution of 5% MCP and FWD, when these disinfectant solutions was removed from the monolayers following 1 h incubation. Only a slight cytotoxic effects was caused by treatment with medical ethanol at 1:10 dilution, and this effect disappeared after the treatment was removed from the monolayers and cells were refed with fresh DMEM with 2% FBS medium overnight (Table 1).

**Table 1:** Tested disinfectants and the cell sensitivity to the disinfectants.

Test products	Active ingredients	Concentrations (v/v)	Dilutions	Cytotoxicity
MCP	Dual quaternary ammonium compounds	5%	1:10	+
			1:100	+
			1:500	−
			1:1000	−
FWD	Dual quaternary ammonium compounds	5%	1:10	+
			1:100	+
			1:500	−
			1:1000	−
Medical EtOH	Ethanol	95%	1:10	+/−*
			1:100	−

“+” means cytotoxicity; “−” means no cytotoxicity.

*A slight cytotoxic effect caused by medical ethanol was observed, but it disappeared after the treatment was removed and cells were refed with fresh DMEM with 2% FBS medium overnight.

### Virucidal efficacy results for Nipah virus

Different disinfectant concentrations and contact periods were evaluated to observe the dose–response and time kinetics of Nipah virus inactivation. We exposed the Nipah virus for 15 s, 30 s, 1 min, 2 min, 4 min and 8 min to two QAC disinfectants at final concentrations ranging from ~ 5% to ~ 0.06% (undiluted to diluted to a 1:81 dilution, a three-fold serial dilution scheme). Each disinfectant tested was effective at inactivating Nipah virus (Table 2).

**Table 2:** Reduction factors (Log_10_) of three disinfectants against Nipah virus.

Test products	Concentrations	Reduction factor (Log_10_)					
		Contact time period					
		15 s	30 s	1 min	2 min	4 min	8 min
MCP	1.67% (5%/3)	> 4	> 4	> 4	> 4	> 4	> 4
	0.56% (5%/9)	> 4	> 4	> 4	> 4	> 4	> 4
	0.19% (5%/27)	1.05	3.17	> 4	> 4	> 4	> 4
	0.06% (5%/81)	0.31	0.61	0.48	0.85	1.25	1.00
FWD	1.67% (5%/3)	> 4	> 4	> 4	> 4	> 4	> 4
	0.56% (5%/9)	> 4	> 4	> 4	> 4	> 4	> 4
	0.19% (5%/27)	> 4	> 4	> 4	> 4	> 4	> 4
	0.06% (5%/81)	0.26	0.6	1.46	1.48	1.94	2.87
MCP-1W	5%	> 4	> 4	> 4	> 4	> 4	> 4
	1.67% (5%/3)	> 4	> 4	> 4	> 4	> 4	> 4
	0.56% (5%/9)	> 4	> 4	> 4	> 4	> 4	> 4
	0.19% (5%/27)	0.79	1.76	> 4	> 4	> 4	> 4
FWD-1W	5%	> 4	> 4	> 4	> 4	> 4	> 4
	1.67% (5%/3)	3.54	> 4	> 4	> 4	> 4	> 4
	0.56% (5%/9)	2.38	> 4	> 4	> 4	> 4	> 4
	0.19% (5%/27)	2.37	3.52	> 4	> 4	> 4	> 4
Medical EtOH (95%)	76%	> 4	> 4	> 4	> 4	> 4	> 4
	57%	> 4	> 4	> 4	> 4	> 4	> 4
	38%	> 4	> 4	> 4	> 4	> 4	> 4
	19%	0.28	0.77	0.96	0.75	2.47	> 4

The virucidal efficacy of the three disinfectants against Nipah virus was addressed by quantitative suspension assay and residual infectivity was determined by virus titre using one-step real-time RT-PCR.

**Figure 1:**
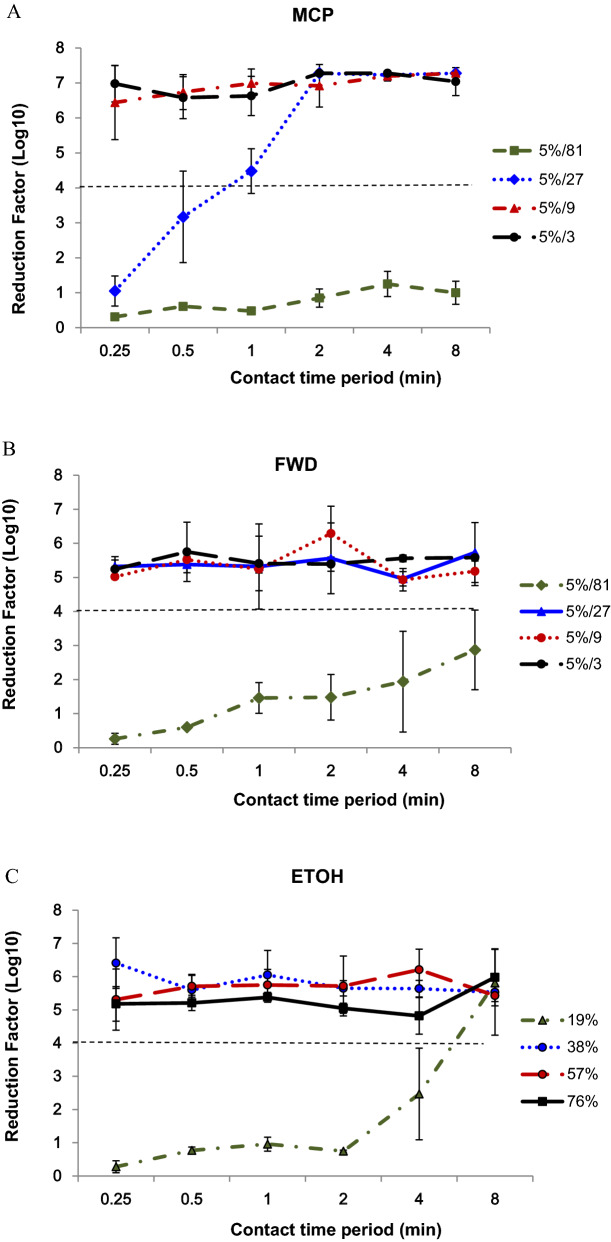
Virucidal activity of MCP (**A**), FWD (**B**) and ethanol (**C**) against Nipah virus. The biocide concentrations ranged from ~ 1.6% to 0.06% (5%/3 to 5%/81, using a three-fold serial dilution scheme for MCP and FWD) or 19% to 76% for ethanol with contact times of 15 s to 8 min. The virucidal efficacy of the three disinfectants against Nipah virus was evaluated by quantitative suspension assay and residual infectivity was determined by virus titer using one-step real-time RT-PCR.

The results show that the dual-QAC disinfectants, MCP and FWD, are highly effective at inactivating Nipah virus within 15 s of contact time, even when they are diluted 1:9 (Table 2 and Fig. 1). The virucidal activity of FWD was found to be superior to that of MCP. For example, FWD diluted up to 27-fold completely inactivate Nipah virus in 15 s, while MCP at the same concentration required 1 min to completely inactivate Nipah virus. Neither of the dual QAC disinfectants completely inactivated Nipah virus when diluted 81 times (5%/81, ~ 0.06% final concentration), even after 8 min of contact time. But the reduction factor (Log_10_) of FWD (5%/81, 8 min) was greater than that of MCP evaluated at the same concentration and contact time, again indicating superiority of FWD over MCP for inactivating Nipah virus.

**Figure 2:**
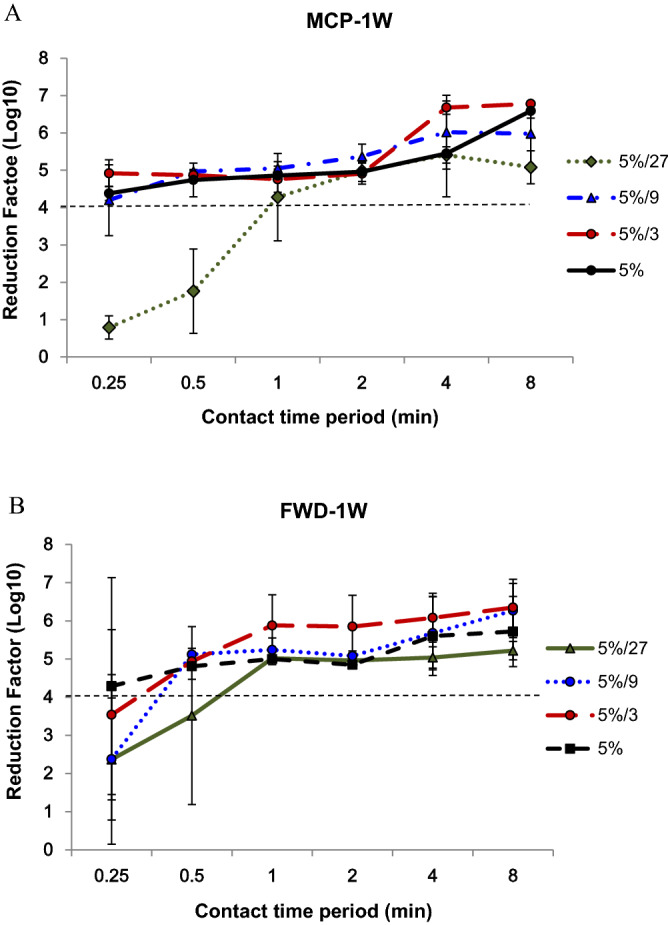
Virucidal activity of undiluted and diluted MCP (**A**) and FWD (**B**) kept for one week at ambient temperature against Nipah virus (MCP-1W and FWD-1W). The biocide concentrations ranged from ~ 5% to 0.19% (5% to 5%/27, using a three-fold serial dilution scheme) with conatct times of 15 s to 8 min. The residual infectivity was determined by virus titer using one-step real-time RT-PCR.

QAC disinfectants usually need to be diluted before use and therefore stability of the virucidal activity in of diluted and undiluted disinfectant needs to be assessed. We therefore assessed the virucidal efficacy of the undiluted QAC or the same disinfectants after diluting and retaining for one week at ambient temperature (RT) (MCP-1W and FWD-1W, Table 2 and Fig. 2). Our results showed that the virucidal efficacy of MCP was similar, and it can be effective against Nipah virus as same as the diluted immediately though the RF of it decreased a little. The virucidal effect of FWD decreased a little after kept for one week at RT, but it can still inactivate the virus when we used the disinfectant with the same concentration and kept longer contact time.

Comparing the results above, we found that the virucidal activity of FWD is little bit better than MCP against Nipah virus, but some effective ingredients to inactivate Nipah virus in FWD are not as very stable as MCP.

The alcohols have rapid bactericidal activity, and are also fungicidal and virucidal, but the degree of effect depends on the percentage concentrations of the alcohol and the physical properties of the target microorganism. Base on previous in vitro studies, 60% to 90% v/v solutions were effective with short contact time and the microbicidal activity of alcohol decreased substantially at concentrations below 50% v/v^24^. However, some studies have shown that ≥ 30% v/v reduced SARS-CoV-2 titers by at least 4 Log_10_ within 30 s^25,26^. None of the publications reported studies of the efficacy of various concentration of ethanol against Nipah virus in detail up to now. Our result showed that 38% ethanol was able to completely inactivate Nipah virus immediately (within 15 s) and 19% ethanol was also able to inactivate Nipah virus but enough contact time is necessary (more than 8 min) in suspension test (Table 2 and Fig. 1C).

Based on the data above we compared the inactivation profiles of three disinfectants against Nipah virus (Table 2). QAC disinfectant (MCP and FWD) exhibit the somewhat dose-dependent inactivating Nipah virus pattern. That is, the better inactivation effect are shown when the disinfectants are used at higher concentration and with longer contact time, and it seemed that enough contact time is more important at the concentration higher than 0.06%, especially when they are diluted and kept for one week.

We used the integrated cell culture real-time PCR method to validate Nipah virus inactivation effect in this study, not to detect the residue virus directly. The general process is same as described in our previous study^25^. Briefly, we pretreated the virus with disinfectants, and then inoculated host cells with the viruses-disinfectant mixture (containing inactivated viruses and infectious viruses). The noninfectious viruses were removed after the viruses-disinfectant mixture dilutions were discarded. Next, the cells were overlaid with fresh medium. Subsequently, the cells were incubated for an optimised period to amplify the intracellular viruses. Finally, CPE was observed 4 days post infection and viral nucleic acids were extracted from the cell culture supernatant and used for one-step real-time RT-PCR with Nipah virus-specific primers to quantify the infectious virus titer after the virus inactivation treatment. The results showed that the CPE of cells infected with virus-disinfectant mixture were consistent with RF results (Table 3).

**Table 3:** CPE of infected cells in each test was observed after virus incubation for different time with different final concentration of MCP, FWD and medical ethanol.

Test products	Concentrations	CPE					
		Contact time period					
		15 s	30 s	1 min	2 min	4 min	8 min
MCP	1.67% (5%/3)	−/−/−	−/−/−	−/−/−	−/−/−	−/−/−	−/−/−
	0.56% (5%/9)	−/−/−	−/−/−	−/−/−	−/−/−	−/−/−	−/−/−
	0.19% (5%/27)	+/+/+	+/−/−	−/+/−	−/−/−	−/−/−	−/−/−
	0.06% (5%/81)	+/+/+	+/+/+	+/+/+	+/+/+	+/+/+	+/+/+
FWD	1.67% (5%/3)	−/−/−	−/−/−	−/−/−	−/−/−	−/−/−	−/−/−
	0.56% (5%/9)	−/−/−	−/−/−	−/−/−	−/−/−	−/−/−	−/−/−
	0.19% (5%/27)	−/−/−	−/−/−	−/−/−	−/−/−	−/−/−	−/−/−
	0.06% (5%/81)	+/+/+	+/+/+	+/+/+	+/+/+	+/+/+	+/+/−
MCP-1W	5%	−/−/−	−/−/−	−/−/−	−/−/−	−/−/−	−/−/−
	1.67% (5%/3)	−/−/−	−/−/−	−/−/−	−/−/−	−/−/−	−/−/−
	0.56% (5%/9)	−/+/−	−/−/−	−/−/−	−/−/−	−/−/−	−/−/−
	0.19% (5%/27)	+/+/+	+/+/+	+/−/−	−/−/−	−/−/−	−/−/−
FWD-1W	5%	+/−/−	−/−/−	−/−/−	−/−/−	−/−/−	−/−/−
	1.67% (5%/3)	−/−/+	−/−/−	−/−/−	−/−/−	−/−/−	−/−/−
	0.56% (5%/9)	+/−/+	−/−/−	−/−/−	−/−/−	−/−/−	−/−/−
	0.19% (5%/27)	+/+/−	+/−/−	−/−/−	−/−/−	−/−/−	−/−/−
Medical EtOH (95%)	76%	−/−/−	−/−/−	−/−/−	−/−/−	−/−/−	−/−/−
	57%	−/−/−	−/−/−	−/−/−	−/−/−	−/−/−	−/−/−
	38%	−/−/−	−/−/−	−/−/−	−/−/−	−/−/−	−/−/−
	19%	+/+/+	+/+/+	+/+/+	+/+/+	−/+/+	−/−/−

We have evaluated the virucidal activity of MCP, FWD and medical ethanol against SARS-CoV-2^25^ and Ebola virus in previous studies, and we determined the virucidal effect of these agents against Nipah virus here. We summarized and compared all of the data in Table 4. Our results show that the three disinfectants displayed efficient inactivation of these challenge emerging viruses: SARS-CoV-2, Ebola virus and Nipah virus (Table 4).

**Table 4:** Summary of reduction factors of three disinfectants against Nipah virus, Ebola virus and SARS-CoV-2.

Test products	Concentrations	Nipah virus		Ebola virus		SARS-CoV-2	
		Contact time period	RF	Contact time period	RF	Contact time period	RF
MCP	5%	ND	ND	> 15 s	> 4	> 15 s	> 4
	1.67% (5%/3)	> 15 s	> 4	> 15 s	> 4	> 15 s	> 4
	0.56% (5%/9)	> 15 s	> 4	> 15 s	> 4	> 15 s	> 4
	0.19% (5%/27)	> 1 min	> 4	> 1 min	> 4	> 1 min	> 4
	0.06% (5%/81)	8 min	< 4	8 min	< 4	8 min	> 4
FWD	5%	ND	ND	> 15 s	> 4	> 15 s	> 4
	1.67% (5%/3)	> 15 s	> 4	> 15 s	> 4	> 15 s	> 4
	0.56% (5%/9)	> 15 s	> 4	> 1 min	> 4	> 15 s	> 4
	0.19% (5%/27)	> 15 s	> 4	> 2 min	> 4	> 30 s	> 4
	0.06% (5%/81)	8 min	< 4	8 min	< 4	8 min	< 4
MCP-1W	5%	> 15 s	> 4	> 15 s	> 4	> 15 s	> 4
	1.67% (5%/3)	> 15 s	> 4	> 4 min	> 4	> 30 s	> 4
	0.56% (5%/9)	> 15 s	> 4	8 min	< 4	> 1 min	> 4
	0.19% (5%/27)	> 1 min	> 4	8 min	< 4	> 2 min	> 4
	0.06% (5%/81)	ND	ND	ND	ND	8 min	> 4
FWD-1W	5%	> 15 s	> 4	> 30 s	> 4	> 15 s	> 4
	1.67% (5%/3)	> 30 s	> 4	8 min	> 4	> 1 min	> 4
	0.56% (5%/9)	> 30 s	> 4	8 min	< 4	> 1 min	> 4
	0.19% (5%/27)	> 1 min	> 4	8 min	< 4	> 2 min	> 4
	0.06% (5%/81)	ND	ND	ND	ND	8 min	< 4
Medical EtOH (95%)	95%	ND	ND	> 15 s	> 4	> 15 s	> 4
	76%	> 15 s	> 4	> 15 s	> 4	> 15 s	> 4
	57%	> 15 s	> 4	> 15 s	> 4	> 15 s	> 4
	38%	> 15 s	> 4	> 15 s	> 4	> 15 s	> 4
	19%	8 min	> 4	8 min	< 4	8 min	< 4

ND: not determined.

As a commercial disinfectant, MCP showed a quite good virucidal activity against the three emerging viruses, and is more stable than FWD which is also a QAC disinfectant. For example, when the diluted FWD with the same concentration was kept for one week at RT, longer contact time was needed to inactivate the viruses. Compared with MCP, FWD is somewhat more effective at inactivating these emerging viruses, especially at the lower concentration against SARS-CoV-2 and Nipah virus. For example, FWD at the concentration of 0.19% (5%/27) inactivated Nipah virus and SARS-CoV-2 quickly (less than 1 min), while MCP at the same concentration took more than 1 min to inactivate these two viruses. Medical ethanol showed similar inactivation profiles against the three emerging viruses. It was surprising that 20% medical ethanol inactivated Nipah virus completely, though more than 8 min of contact time was required in this case.

These data suggest that inactivation of Ebola virus may be more difficult than inactivation of SARS-CoV-2 and Nipah virus. The FWD with the same concentration (diluted 9 × or more) needed more contact time to inactivate Ebola virus than SARV-CoV-2 and Nipah virus, and the virucidal activity of diluted MCP kept for one week at RT (MCP-1W, dilute 3 × or more) showed a similar result. This indicates that these QAC disinfectants should be diluted freshly for Ebola virus inactivation. Comparatively speaking, Nipah virus may be the most susceptible virus among these emerging viruses to inactivation by the three disinfectants.

## Discussion

As an increasingly global pandemic threat with high case fatality rate, wide host range and expanding modes of transmission coupled with the absence of effective vaccine or therapeutic agents, WHO have declared Nipah virus a priority pathogen for the research and development of diagnostic, prevention and treatment strategies. This virus, has also been included as a priority for vaccine development by the Coalition for Epidemic Preparedness Innovations (CEPI)^6^. A high rate of inter-person transmission with no intermediate hosts has been reported recently in Bangladesh and India outbreaks^27^. What’s more, recurring Nipah virus outbreaks have been reported almost yearly throughout South and Southeast Asia since 2001^28^. All the information cautions us that continued researches into antiviral drug therapies and vaccines are necessary, as are more comprehensive public health measures comprising a combination of education, hygiene and necessary practices to prevent potentially future outbreaks. Use of an effective virucidal agent for disinfecting objects contaminated with Nipah virus represents an important intervention, thereby mitigating the risk of virus transmission. However, only a few related data for Nipah virus could be identified during this literature search^22^.

In this study, we evaluated in detail the virucidal efficacy of two QAC disinfectants (considered low-level disinfectants), against Nipah virus. Our results showed that MCP (approved by EPA) and a novel QAC disinfectant (FWD) displayed > 4 Log_10_ inactivation of Nipah virus though the virucidal efficacy between MCP and FWD varied slightly. The slight differences in efficacy noted may be due to the different compositiosn of these QAC. As we known, QACs are cationic detergents and the cation portion consists of the central nitrogen with four attached groups, which occur in a variety of structures. These variations can affect the antimicrobial activity of the QAC in terms of dose and action against different groups of microorganisms^29^. Despite the inactivation effectiveness for viruses, the potential environmental impact of QACs also should be considered. As a novel disinfectant based on dual quaternary ammonium compounds, FWD lacks surfactants known as nonylphenol ethoxylates (NPE) that are considered highly toxic to the aquatic environment. Though some effective ingredients in FWD are not as stable as those in MCP, aged FWD remains effective against Nipah virus albeit with longer contact time. These results indicate that FWD may could be considered as a potential alternate for MCP.

The ongoing COVID-19 pandemic caused by SARS-CoV-2 has drawn broader attention and initiated widespread academic research with the goal of controlling the virus and the disease including various decontamination measures for environment and population. Disinfectants may play important roles in defense against the emerging viruses directly. We report bench-scale experiments evaluating the virucidal activity of three disinfectants against Nipah virus, compared with efficacy for inactivating SARS-CoV-2 and Ebola virus in quantitative suspension test. Two QAC disinfectants (MCP and FWD) and Medical EtOH each display rapid virucidal activity, but further inactivation experiments may have to be repeated using downstream application-specific matrices. As a continually evolving area, new disinfectant product formulations are constantly appearing in the market to meet challenges posed by emerging pathogens. Each disinfectant has its advantages and disadvantages for a particular situation. These novel disinfectants need to be evaluated before use.

Efficacy of disinfectants is dependent on the target organism(s)^26,30^. The inactivation profiles of the three evaluated disinfectants against different enveloped viruses were similar, as might be expected. Certain differences in efficacy were discovered. For example, MCP-1W (kept for one week at RT) at the concentration of about 0.56% was still able to inactivate SARS-CoV-2 and Nipah virus, but not Ebola virus after more than 8 min contact time, though all of them are enveloped virus with single-stranded RNA genome. It is important to note that the data related to the virucidal activity of some disinfectants which were determined by surrogate virus such as bacteriophages, enveloped viruses, or non-enveloped viruses should not be extrapolated for use with these emerging viruses without further proof. Owing to their high lethality, Ebola virus and Nipah virus are generally classified as biosafety level-4 (BSL-4) pathogens, and testing conducted specifically with these emerging viruses are needed to confirm expected results.

In this study, we utilized integrated cell culture real-time quantitative RT-PCR method to assess the virucidal efficacy of disinfectants and addressed the advantage of this method further^31–33^. This method utilizes the host cell as an efficient tool to separate infectious and noninfectious viruses, and to enable the amplification only of viruses capable of infecting the host cell. Accompanied with real-time RT-PCR, it decreases the limit of quantitation and improves the sensitivity of detection. This method even could be used to evaluate the possibility of virus being present at levels lower than the limit of detection of a TCID_50_/plaque assay. And higher log inactivation values might be possible without limitations in the amount of challenge virus that can be applied. For example, when the initial titre of the virus used for the inactivation effect test is low, RF > 4 cannot be reached^34^. Hence our method ensured that the smallest amount of possibly non-inactivated virus could be detected, and a very long passaging time and/or large quantity of culture was not needed.

Even in the long-established fields of application, one can expect more effective, mild and eco-friendly disinfectant or virucidal agents to be employed in the various applications further. One aim of our study was to provide a summary that bridge between interested scientists from different disciplines including chemistry, biology and public health etc. By designing tailor-made disinfectants or advanced formulations, public health experts can expect more and accurate choice of disinfectants for decontamination in healthcare settings as part of infection prevention and control for emerging infectious diseases.

Finally, the emerging pathogens, such as Nipah virus, Ebola virus and SARS-CoV-2, are limited to handling at high levels of biosafety containment (BSL4 and BSL-3). Use of validated methods of disinfection is an essential requirement when working with these emerging viruses in BSL-4 and BSL-3. This study may assist in disinfectant choice and in developing and improving related operating procedures for high levels of biosafety laboratories.

## Methods

### Virucidal products tested

In this study, the virucidal efficacies of three disinfectants against Nipah virus, Micro-Chem Plus® (MCP, National Chemical Laboratories, Inc., Philadelphia, Pennsylvania), FWD and Medical EtOH, were evaluated in suspension tests according to the Technical Standard for Disinfection of China^23^. Among these disinfectants, Medical EtOH and Micro-Chem Plus® are commercial, broad-spectrum disinfectants. FWD (Xinxiang Dashin Forest Technology Co., Ltd) is a dual quaternary ammonium compound (QAC) product similar to MCP, and is still in the research and development stage.

### Cell culture and virus strain

The Vero E6 cells were obtained from the Preservation Center in Wuhan Institute of Virology, Chinese Academy of Sciences, and were cultured at 37 °C with 5% CO_2_ in Dulbecco’s Modified Eagle medium (DMEM) supplemented with fetal bovine serum (FBS). Nipah virus—Bangladesh, Ebola virus-Mayinga and SARS-CoV-2 (SARS-CoV-2 China/Wuhan/WIV04/201912) were provided by National Biosafety Laboratory, Chinese Academy of Sciences, and was propagated in Vero E6 cells. All work involving infectious Nipah virus, Ebola virus and SARS-CoV-2 were performed in BSL-4 facility in National Biosafety Laboratory, Wuhan.

### Determination of cytotoxicity

The mixtures of viruses and disinfectants were diluted before testing to reduce the potential cytotoxicity. Cytotoxic effects were first assessed in Vero E6 cells using medium (DMEM with 2% FBS) and disinfectant only to ensure that diluted disinfectants was not cytotoxic, as described previously^25^. Briefly, the test products were serially diluted, and 1 ml dilution from each sample was inoculated into Vero E6cells. After 1 h of incubation at 37 °C, dilutions were discarded, and cells were overlaid with fresh medium and cultured for 4 days. The cells were observed daily for cytotoxic effects by light microscopy.

### Virus titration

Nipah viruses were titrated by TCID_50_ on Vero E6 cells using the protocol described previously^25^. Briefly, 96-well plates containing Vero E6 cells were incubated for 1 h at 37 °C in a 5% CO_2_ incubator with 0.1 ml of serial dilutions of virus stocks using 1:10 as the starting dilution. Then 0.1 ml of 2% FBS medium was added to each well. After incubation for 4 days at 37 °C in 5% CO_2_, CPE was observed and used to calculate the virus titer in TCID_50_/ml.

### Inactivation assay using quantitative suspension Test

As described in our previous study^25^, equal volumes of disinfectant at different concentration and Nipah virus stock were mixed for each of the inactivation experiments. Immediately after incubation for defined periods of time (such as 15 s, 30 s, 1 min, 2 min, 4 min and 8 min) at room temperature, the mixture were diluted with a large amount of medium to quench the inactivation reaction and eliminate the toxicity of disinfectant. Some samples were taken out from each dilution to infect cells. After incubating for 1 h at 37 °C in a 5% CO_2_ incubator, the viruses-disinfectant mixture dilutions were discarded. Next, the cells were overlaid with fresh medium. The CPE was observed after 4 days post infection and Nipah viral RNA was extracted from the supernatant. Quantitative one-step real-time RT-PCR was used to assess virus titer. For each experiment, virus control containing medium instead of disinfectant was included, and each experiment was repeated three times.

### Extraction of viral RNA and quantitative RT-PCR

**Figure 3:**
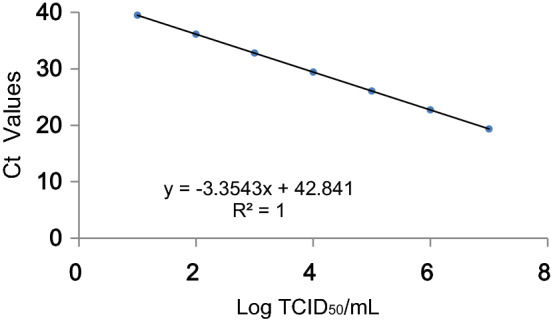
One-step real-time RT-PCR standard curve for Nipah virus. The virus stock was serial diluted (1:10) and viral RNAs were extracted from these serial dilutions. Ct values of the range of dilutions covering 7 Log_10_ were used to draw the standard curve. The corresponding virus titre was calculated based on the standard curve.

Nipah viral RNA was extracted from 140 μl of supernatant from virus-infected Vero E6 cells using the RNA extraction kit (QIAamp Viral RNA Mini Kit, Qiagen Inc., Valencia, CA, USA) following the manufacturer’s instructions. The extracts were resuspended in 60 μl of Buffer AVE, aliquoted and stored at − 70 °C before RT-PCR amplification was carried out. Viral RNA was quantified by One-step real-time RT-PCR using using HiScript II One Step qRT-PCR Probe Kit (Vazyme) according to the manufacturer’s instructions and with the modified Nipah virus specific assay-NiV-NP-UD (NiV-NP-U: GCAGGATTCTTCGCAACCAT; NiV-NP-D: TGGTGTTGAGGTCACTCTGGA; NiV-NP-P: FAM-CAAGTGCTGGATACCTTGTCTCCAACCC-BHQ1)^35^. The standard curve for Nipah virus titres calculation was established using the Nipah virus stock with known titres (Fig. 3). The virus stock was serial diluted (1:10) and viral RNAs were extracted from these serial dilutions. Ct values of the range of dilutions covering 7 Log_10_ were used to draw the standard curve. The corresponding virus titre was calculated based on the standard curve.

### Calculation of the reduction factor (RF)

According to the standard requirement^23^, the virucidal activity was determined by the difference of the logarithmic titre of the virus control minus the logarithmic titer of the test virus, reduction factor (RF). The Log10 titer and its standard deviation (SD, n = 3) were calculated as well as the variance of the RF. RF of ≥ 4 log10 was regarded as evidence of efficient virucidal activity.

## Supplementary Information

Supplementary Table 1.

## Data Availability

The datasets used and/or analyzed during the current study are available from the corresponding author on reasonable request, and partial data analysed during this study are included in this published article and its supplementary information files: Huang Y, Xiao S, Song D, Yuan Z. Evaluating the virucidal activity of four disinfectants against SARS-CoV-2. Am J Infect Control. 2022 Mar;50(3):319–324. 10.1016/j.ajic.2021.10.035, and we have cited it in the manuscript.
