# Supplementary material for: Evaluation and comparison of three virucidal agents on inactivation of Nipah virus
Source: Sci Rep. 2022 Jul 5;12:11365. doi: 10.1038/s41598-022-15228-0 (PMC9255448; doi:10.1038/s41598-022-15228-0)
Supplement: Supplementary file 1 — Supplementary Table 1. [file 41598_2022_15228_MOESM1_ESM.docx]

**Supplementary**

**Table 1.** Raw data of reduction factors of three disinfectants against Nipah virus.

| **Test products** | **Concentrations** | **Reduction factor** | | | | | |
| --- | --- | --- | --- | --- | --- | --- | --- |
|  |  | **Contact time period** | | | | | |
|  |  | **15 sec** | **30 sec** | **1 min** | **2 min** | **4 min** | **8 min** |
| MCP | 1.67% (5%/3) | 6.98 | 6.58 | 6.63 | 7.28 | 7.28 | 7.04 |
|  | 0.56% (5%/9) | 6.44 | 6.74 | 6.99 | 6.92 | 7.2 | 7.28 |
|  | 0.19% (5%/27) | 1.05 | 3.17 | 4.48 | 7.28 | 7.23 | 7.28 |
|  | 0.06% (5%/81) | 0.31 | 0.61 | 0.48 | 0.85 | 1.25 | 1.00 |
| FWD | 1.67% (5%/3) | 5.24 | 5.75 | 5.41 | 5.39 | 5.56 | 5.58 |
|  | 0.56% (5%/9) | 5.02 | 5.52 | 5.24 | 6.29 | 4.93 | 5.18 |
|  | 0.19% (5%/27) | 5.32 | 5.38 | 5.32 | 5.56 | 4.96 | 5.73 |
|  | 0.06% (5%/81) | 0.26 | 0.6 | 1.46 | 1.48 | 1.94 | 2.87 |
| MCP-1W | 5% | 4.38 | 4.74 | 4.86 | 4.96 | 5.45 | 6.59 |
|  | 1.67% (5%/3) | 4.92 | 4.87 | 4.76 | 4.91 | 6.68 | 6.78 |
|  | 0.56% (5%/9) | 4.2 | 4.97 | 5.05 | 5.37 | 6.02 | 5.98 |
|  | 0.19% (5%/27) | 0.79 | 1.76 | 4.28 | 4.98 | 5.41 | 5.08 |
| FWD-1W | 5% | 4.29 | 4.81 | 5 | 4.85 | 5.6 | 5.72 |
|  | 1.67% (5%/3) | 3.54 | 4.94 | 5.88 | 5.85 | 6.08 | 6.35 |
|  | 0.56% (5%/9) | 2.38 | 5.12 | 5.24 | 5.08 | 5.68 | 6.27 |
|  | 0.19% (5%/27) | 2.37 | 3.52 | 5.02 | 4.96 | 5.04 | 5.22 |
| Medical EtOH  (95%) | 76% | 5.18 | 5.21 | 5.38 | 5.05 | 4.82 | 5.98 |
|  | 57% | 5.31 | 5.71 | 5.75 | 5.72 | 6.21 | 5.43 |
|  | 38% | 6.41 | 5.6 | 6.05 | 5.65 | 5.64 | 5.53 |
|  | 19% | 0.28 | 0.77 | 0.96 | 0.75 | 2.47 | 5.81 |
